# Development and validation of Comprehensive Gait Assessment using InerTial Sensor score (C-GAITS score) derived from acceleration and angular velocity data at heel and lower trunk among community-dwelling older adults

**DOI:** 10.1186/s12984-019-0539-3

**Published:** 2019-05-28

**Authors:** Shogo Misu, Tsuyoshi Asai, Takehiko Doi, Ryuichi Sawa, Yuya Ueda, Shunsuke Murata, Takashi Saito, Taiki Sugimoto, Tsunenori Isa, Yamato Tsuboi, Minoru Yamada, Rei Ono

**Affiliations:** 1 Department of Physical Therapy, Faculty of Nursing and Rehabilitation, Konan Women’s University, 6-2-13, Morikita-machi, Higashinada-ku, Kobe, 658-0001 Japan; 2 Department of Community Health Sciences, Kobe University Graduate School of Health Sciences, 7-10-2 Tomogaoka, Suma-ku, Kobe, 654-0142 Japan; 3 Department of Physical Therapy, Faculty of Rehabilitation, Kobegakuin University, 516 Arise, Ikawadani-cho, Nishi-ku, Kobe, 651-2180 Japan; 4 Department of Preventive Gerontology, Center for Gerontology and Social Science, National Center for Geriatrics and Gerontology, 35 Gengo Morioka, Obu, 474-8511 Japan; 5 Japan Center for International Exchange, Meisan Tameike Bldg. 7F, 1-1-12 Akasaka, Minato-ku, Tokyo, 107-0052 Japan; 6 Japan Society for the Promotion of Science, Research Fellowship for Young Scientists, Kojimachi Business Center Building, 5-3-1 Kojimachi, Chiyoda-ku, Tokyo, 102-0083 Japan; 7 The Center for Comprehensive Care and Research on Memory Disorders, National Center for Geriatrics and Gerontology, Obu, Japan; 8 Medical Genome Center, National Center for Geriatrics and Gerontology, Obu, Japan; 9 Graduate School of Comprehensive Human Sciences, University of Tsukuba, Tokyo, Japan, 3-29-1 Otsuka, Bunkyo-ku, Tokyo, 112-0012 Japan

**Keywords:** Gait, Score, Validity, Inertial sensor, Acceleration, Angular velocity, Community-dwelling older people

## Abstract

**Background:**

Although some gait parameters from inertial sensors have been shown to be associated with important clinical issues, because of controversial results, it remains uncertain which parameters for which axes are clinically valuable. Following the idea that a comprehensive score obtained by summing various gait parameters would sensitively reflect declines in gait performance, we developed a scoring method for community-dwelling older adults, the Comprehensive Gait Assessment using InerTial Sensor score (C-GAITS score). The aim of this study was to examine the internal consistency and the construct validity of this method.

**Methods:**

In this cross-sectional study, the gait performance of 378 community-dwelling older people (mean age = 71.7 ± 4.2 years, 210 women) was assessed using inertial sensors attached to the heel and lower trunk. Participants walked along a 15-m walkway, and accelerations, angular velocity, and walking time were measured. From these data, walking speed, mean stride time, coefficients of variation of stride time and swing time, and autocorrelation coefficients and harmonic ratios of acceleration in vertical, mediolateral, and anteroposterior directions at the lower trunk were calculated. Scoring was performed based on quartile by gender (i.e., scored from 0 to 3) for each of the 10 gait parameters. The C-GAITS score was the sum of these scores (range: 0–30). Lower extremity strength, balance function, fall history, and fear of falling were also assessed.

**Results:**

An exploratory factor analysis revealed that the C-GAITS score yielded four distinct factors explaining 57.1% of the variance. The Cronbach’s alpha coefficient was 0.77. A single linear regression analysis showed a significant relationship between total C-GAITS score and walking speed (adjusted *R*^2^ = 0.28). Results from bivariate comparisons using unpaired *t*-tests showed that the score was significantly related to age (*p* = 0.002), lower extremity strength (*p* = 0.007), balance function (*p* <  0.001), fall history (*p* = 0.04), and fear of falling (*p* <  0.001).

**Conclusions:**

Good internal consistency and appropriate construct validity of the C-GAITS score were confirmed among community-dwelling older adults. The score might be useful in clinical settings because of ease of use and interpretation and capability of capturing functional decline.

## Background

Gait is a major physical activity in daily life. Taking more steps leads to higher levels of functional health among older adults, and gait ability is thus an important factor for an independent and healthy life [1–5]. Therefore, the importance of the assessment of gait performance is widely accepted. Walking speed is the most commonly used parameter to assess gait performance and has been reported to be a predictor of functional decline and survival [2, 3]. Postural control during gait requires a complex neuromuscular system. The decline of this system with aging makes postural control a challenging task, resulting in older adults tending to have problems with gait safety and efficiency [6–10]. Thus, objective assessments of various aspects of the quality of gait movement provide a great deal of additional information about gait performance.

Inertial sensors have been proposed as a method for assessing the quality of gait movement. These are appropriate for gait assessment in clinical settings because of their low cost, small size, and durable structure [11]. Gait parameters calculated using acceleration or angular velocity data measured by inertial sensors attached to the lower extremities or trunk have been reported in many studies [4, 11, 12]. Previous studies have reported associations between these gait parameters and important clinical issues, such as aging, risk of falling, and functional declines [13–18]. Temporal parameters such as stride time or swing time represent pace of movement and balance control during gait [19]., and their variability is a marker of consistent and steady gait movement [20]. Gait parameters based on trunk movement (e.g., the regularity and smoothness of trunk acceleration) are also important, because the center of mass is in the lower trunk and the trunk plays an essential role in providing a stable platform for vision and head control [21].

Although the abovementioned gait parameters are associated with many important clinical issues, the assessment of gait using inertial sensors does not seem to have been widely applied in clinical settings. This approach is considered to have several problems from the clinical view, including a lack of clarity about which parameters for which axes are clinically valuable because past studies have yielded controversial results [13, 14, 16, 18, 22, 23]. Various abnormal gait patterns are exhibited by older adults because of complex postural control systems [24]. Therefore, we considered that multiple abnormal gait patterns may overlap among older adults with severe gait problems and expected that a comprehensive score obtained by summing the scores from various gait parameters would sensitively reflect declines in gait performance. In other words, the variability of abnormal gait patterns might explain the controversial results from past studies mentioned above. However, comprehensive scoring methods have not previously been reported, and the usefulness of this approach remains unclear. In addition, adequate reference data from healthy older adults are limited, especially in terms of measurements taken by inertial sensors at the trunk and lower extremities among community-dwelling older adults. Scoring based on these data will be helpful for judging whether obtained data are “good” or “bad” in clinical settings.

In this study, we developed a scoring method to comprehensively assess gait performance by incorporating and summing the scores from various gait parameters using inertial sensors among community-dwelling older adults. This scoring method is the Comprehensive Gait Assessment using InerTial Sensor score (C-GAITS score). The C-GAITS score includes assessment measures for temporal gait parameters and their variability, as well as parameters for control of trunk movement. The aim of this study was to examine the internal consistency and the construct validity of the C-GAITS score.

## Methods

### Subjects

This study used a cross-sectional design. We recruited 463 community-dwelling older adults through a community organization for older adults in the city of Himeji from September 2011 to December 2015. The measurements were collected annually over a period of one or two days, and different older adults participated in each year. The sample size was determined by how many older adults joined the organization and participated in the measurements. Eligibility criteria for this study were being over 65 years and under 85 years old (because the effects of aging on gait become significantly stronger after the age of 85 years [25].) and having the ability to walk independently without an assistive device (because walking with an assistive device significantly changes gait patterns). A total of 434 participants met both eligibility criteria. Participants were excluded if they had a self-reported history of neuromuscular disease that clearly affected gait, such as stroke or Parkinson’s disease (*n* = 6). Participants with cognitive impairments assessed by a trained physical therapist were also excluded (presenting with either a Rapid Dementia Screening Test score < 7 [26]. or a Mini Mental State Examination score < 24 [27].; *n* = 42). In addition, participants who did not complete our assessment of gait performance were excluded (*n* = 8). The final sample for the analysis included 378 participants. Background characteristics were assessed using a questionnaire that included questions on age, gender, and medical conditions (hypertension, diabetes mellitus, heart disease, and respiratory disease [yes/no]). Anthropometric indices (height and weight) were obtained by physical examination. Body mass index was calculated by dividing weight in kilograms by height in meters squared (kg/m^2^). This study was carried out in accordance with the principles of the Declaration of Helsinki. The Research Ethics Committee of Kobe Gakuin University approved the study (Approval No. HEB100806–1). Informed consent was obtained from all participants prior to participation.

### Gait assessment

Participants walked along a smooth, horizontal walkway 15 m in length. Before the gait assessment, it was ensured that all participants were wearing appropriately sized shoes. A 10-m section of the walkway was marked with two lines positioned 2.5 m from either end. This was to allow for adequate space and time for acceleration and deceleration. After familiarization with the walkway, the participants were instructed to walk at a self-selected, preferred speed. An assistant walked beside the participant to prevent falls and to measure the participant’s walking time in the middle 10-m section of the walkway with a stopwatch. Walking speed was expressed in meters per second (m/s). This method has been reported to be as accurate as automated measurements [28].

Heel and trunk acceleration and angular velocity while walking were measured using two miniature, wireless inertial sensors (MVP- RF8; MicroStone, Nagano, Japan) with a sampling rate of 200 Hz. One sensor was attached to the posterior surface of the participant’s right heel with surgical tape, and the other was fixed to a belt at the level of the L3 spinous process. In this way, acceleration and angular velocity could be measured without restricting movement. All signals were synchronously and wirelessly transferred to a personal computer through a Bluetooth personal area network.

Signal processing was performed with commercially available software (MATLAB, Release 2011b; The Math Works Japan, Tokyo, Japan). Acceleration and angular velocity data were first sent through a low-pass filter with a cutoff frequency of 20 Hz. Based on our previous work, heel-contact events were identified as vertical acceleration peaks of the heel, and toe-off events were identified as the time of the first maximum value of the sagittal angular velocity after quiet standing and every other maximum [29]. The following gait parameters were based on data acceleration and angular velocity data from five consecutive strides during steady-state walking to exclude data from the first two consecutive strides.

Mean stride time and mean swing time were computed by using heel-contact and toe-off events. Mean swing time is presented as a ratio of stride time (% swing time), reflecting the percent of the period accounted for by the single-limb support phase, which is associated with balance control [19]. Additionally, to estimate the variability of gait, we calculated the coefficient of variation (CV) of stride time and of swing time using the following formula: CV = (standard deviation/mean) × 100.

To evaluate the regularity of trunk movement, acceleration data of the trunk for each direction, namely vertical (VT), mediolateral (ML), and anteroposterior (AP), were analyzed using an unbiased autocorrelation procedure that has been described elsewhere [30]. In brief, an unbiased autocorrelation coefficient (AC) is an estimate of the regularity of a time series by cross-correlation with itself at a given time shift; it is independent of the amount of data managed. A perfect replication of the gait cycle signal between neighboring strides would return an AC of one, and no association would yield a coefficient of zero.

The harmonic ratio (HR) was used to evaluate the control of trunk oscillation, indicating the walking smoothness during gait and step-to-step asymmetry [31, 32]. The theory behind HR and its calculation has been described previously [31]. In brief, digital Fourier transforms were performed on the acceleration signals of each stride at the trunk. The HRs in the VT and AP directions were calculated as the sum of the amplitudes of the first 10 *even* harmonics divided by the sum of the amplitudes of the first 10 *odd* harmonics. In contrast, the HR in the ML direction was calculated as the sum of the amplitudes of the *odd* harmonics divided by the sum of the amplitudes of the *even* harmonics. The means of the calculated HRs were used in the analyses. Higher HR values indicated greater walking smoothness, and lower HR values indicated less smooth movement.

### The comprehensive gait assessment using InerTial sensor (C-GAITS) score

Development of the C-GAITS score was performed using the following gait parameters:

- walking speed
- mean stride time, % swing time
- CV of stride time, CV of swing time
- AC in the VT, ML, and AP directions
- HR in the VT, ML, and AP directions

To equalize the contribution of each gait parameter to the score, scoring was performed according to the quartile rank by gender for each gait parameter (i.e., lower than the first quartile was scored 0, between the first quartile and the median was scored 1, between the median and the third quartile was scored 2, and higher than the third quartile was scored 3; scores ranged from 0 to 3). The C-GAITS score was calculated by summing these scores.

### Clinical assessments

We administered the five-chair-stand test (5CS) and the tandem balance test (tandem test) to assess lower extremity strength and balance function, respectively. In the 5CS, participants were asked to stand up from a chair and then sit down five times as quickly as possible while keeping their arms folded across their chests. Participants who took over 11.1 s to complete the 5CS was classified as having impaired lower-extremity strength [33]. In the tandem test, participants were requested to hold the tandem position for 10 s with eyes open, and the time was measured until participants moved their feet or grasped the measurer for support, or until the 10 s had elapsed. Participants who held the tandem position for less than 3 s in the tandem test were classified as having impaired balance [33]. Fall history in the previous year and the psychological state of having a fear of falling (FoF) were also assessed using self-administered questionnaires. A fall was defined as “an event that resulted in the participant unintentionally coming to the ground or other lower level” [34]. FoF was assessed using the question “Are you afraid of falling (yes/no)?” [35].

### Statistical analysis

Background characteristics and gait parameters were compared by gender using unpaired *t*-tests or *χ*^2^ tests.

An exploratory factor analysis for the C-GAITS score was conducted to assess the structural validity and subdomain of construct validity. The extraction method was the unweighted least squares procedure, and the number of factors was based on eigenvalues > 1. The factors were expected to be correlated, so promax rotation used. Items that met a minimum loading of 0.5 were considered relevant, and only these gait parameters were ultimately incorporated into the C-GAITS score. According to the results of the factor analysis, each subscale score was calculated by summing the scores included in each factor.

Cronbach’s alpha coefficient was used to determine the internal consistency of the C-GAITS score. The coefficient was calculated from the gait parameter scores incorporated in the C-GAITS score. An alpha coefficient of 0.70 was considered to indicate good internal reliability [36].

To further assess the construct validity, hypothesis testing was performed. A single linear regression analysis was performed between the total C-GAITS score and walking speed. Additionally, unpaired *t*-tests were performed using the total C-GAITS score and the subscale scores derived from the factor analysis as dependent variables. Age (older-old adults: age ≥ 75 years vs. younger-old adults: age < 75 years), lower-extremity strength, balance function, fall history, and fear of falling were used as group variables. We hypothesized that the C-GAITS score is correlated with walking speed. Additionally, the C-GAITS score was hypothesized to be associated with age, lower extremity strength, balance function, fall history, and FoF. Walking speed is a measure that represents gait function, so this measure was expected to be correlated with the other gait parameters. Theoretically, poor gait performance reflects older age, poorer lower extremity strength, and reduced balance function and leads to high falling risk and having FoF.

All statistical analyses were carried out using commercially available software (IBM SPSS statistics software, Version 20; IBM Corp., Armonk, NY, USA). The level of significance for all analyses was set at 5%.

## Results

The participants’ characteristics and gait performance measures are summarized in Table 1. The mean age (± standard deviation [SD]) was 71.7 ± 4.2 years. There were 210 women (55.6%) and 168 men (44.4%). Compared with men, women were older (*p* = 0.02), and the women’s group included more participants who had fallen in the previous year (*p* = 0.003) and more individuals with FoF (*p* <  0.001). In the gait assessment results, compared with men, women presented a significantly slower mean stride time (women: 0.95 ± 0.07 s, men: 1.02 ± 0.08 s, p <  0.001), a higher mean value of AC-ML (women: 0.70 ± 0.15, men: 0.67 ± 0.13, *p* = 0.03), a higher mean value of HR-ML (women: 2.38 ± 0.69, men: 2.11 ± 0.60, p <  0.001), and a higher mean value of HR-AP (women: 3.87 ± 1.02, men: 3.39 ± 0.94, p <  0.001). No significant differences between genders were seen in walking speed (women: 1.41 ± 0.18 m/s, men: 1.40 ± 0.21 m/s, *p* = 0.67) or the other gait performance measures.

**Table 1:** Characterictics and gait parameters of participants by gender

Variable	Total	Women	Men	*p-*value
	(*n* = 378)	(*n* = 210, 55.6%)	(*n* = 168, 44.4%)	
Age (in years)	71.7 ± 4.2	72.1 ± 4.4	71.3 ± 3.9	0.08
Weight (in kilograms)	57.8 ± 9.8	52.8 ± 7.7	64.1 ± 8.5	< 0.001
Height (in meters)	1.57 ± 0.09	1.51 ± 0.05	1.64 ± 0.06	< 0.001
Body mass index (in kg/m^2^)	23.4 ± 2.9	23.2 ± 3.0	23.7 ± 2.8	0.14
Medical history, n (%)				
Hypertension	83 (40.3)	83 (40.3)	70 (42.4)	0.75
Diabetes	18 (8.7)	18 (8.7)	19 (11.5)	0.39
Heart disease	18 (8.7)	18 (8.7)	23 (13.9)	0.13
Respiratory disease	14 (6.8)	14 (6.8)	9 (5.5)	0.67
Older-old adult (aged ≥75 years), n (%)	96 (25.4)	63 (30.0)	33 (19.6)	0.02
Impaired lower-extremity strength (5CS > 11.1 s), *n* = 376, n (%)	43 (11.4)	20 (9.6)	23 (13.7)	0.25
Impaired balance (tandem test < 3 s), *n* = 369, n (%)	38 (10.3)	24 (11.7)	14 (8.5)	0.39
Fall history in the previous year (faller), *n* = 377, n (%)	65 (17.2)	47 (22.5)	18 (10.7)	0.003
FoF (yes), *n* = 372, n (%)	96 (25.8)	78 (37.7)	18 (10.9)	< 0.001
Walking speed (in meters/second)	1.40 ± 0.20	1.41 ± 0.18	1.40 ± 0.21	0.67
Mean stride time (in seconds)	0.98 ± 0.08	0.95 ± 0.07	1.02 ± 0.08	< 0.001
% swing time	41.9 ± 2.3	41.9 ± 2.2	41.9 ± 2.4	0.94
CV of stride time, %	1.97 ± 1.00	1.97 ± 1.03	1.98 ± 0.95	0.87
CV of swing time, %	3.11 ± 1.54	3.04 ± 1.49	3.20 ± 1.60	0.29
AC-VT	0.86 ± 0.09	0.86 ± 0.10	0.86 ± 0.08	0.77
AC-ML	0.69 ± 0.14	0.70 ± 0.15	0.67 ± 0.13	0.03
AC-AP	0.85 ± 0.09	0.86 ± 0.10	0.85 ± 0.08	0.40
HR-VT	3.37 ± 0.87	3.40 ± 0.87	3.33 ± 0.88	0.44
HR-ML	2.26 ± 0.67	2.38 ± 0.69	2.11 ± 0.60	< 0.001
HR-AP	3.66 ± 1.01	3.87 ± 1.02	3.39 ± 0.94	< 0.001

Values are means ± standard deviations or percentages. *P-*values were calculated using unpaired *t-*tests or *χ*^2^ tests by gender

FoF: fear of falling, 5CS: five-chair-stand test; tandem test: tandem stand test, CV: coefficient of variation, AC: autocorrelation coefficient, VT: vertical, ML: mediolateral, AP: anteroposterior, HR: harmonic ratio

The reference values for the scoring of the C-GAITS based on the quartiles for each gait parameter are presented in Table 2. The % swing time score was excluded from the calculation of the C-GAITS score because of its low factor loading (< 0.5) in the exploratory factor analysis. Consequently, the remaining 10 items were selected as items for the C-GAITS score. The mean total score was 15.0 ± 6.4 (range: 0–30).

**Table 2:** Reference values based on quartiles for each gait parameter in the Comprehensive Gait Assessment using InerTial Sensor (C-GAITS) score

	Score	Walking speed	Mean stride time	CV of stride time	CV of swing time	AC-VT	AC-ML	AC-AP	HR-VT	HR-ML	HR-AP
Women	3	≥ 1.54	≤ 0.89	≤ 1.20	≤ 1.80	≥ 0.93	≥ 0.82	≥ 0.93	≥ 3.88	≥ 2.75	≥ 4.37
	2	1.43–1.53	0.90–0.93	1.21–1.72	1.81–2.87	0.89–0.92	0.72–0.81	0.88–0.92	3.32–3.87	2.37–2.74	3.76–4.36
	1	1.28–1.42	0.94–0.98	1.73–2.54	2.88–3.88	0.82–0.88	0.61–0.71	0.81–0.87	2.79–3.31	1.88–2.36	3.16–3.75
	0	≤ 1.27	≥ 0.99	≥ 2.55	≥ 3.89	≤ 0.81	≤ 0.60	≤ 0.80	≤ 2.78	≤ 1.87	≤ 3.15
Men	3	≥ 1.53	≤ 0.95	≤ 1.37	≤ 2.00	≥ 0.92	≥ 0.77	≥ 0.91	≥ 3.85	≥ 2.43	≥ 3.88
	2	1.40–1.52	0.96–1.01	1.38–1.69	2.01–2.89	0.88–0.91	0.67–0.76	0.86–0.90	3.16–3.84	1.99–2.42	3.25–3.87
	1	1.25–1.39	1.02–1.06	1.70–2.40	2.90–4.20	0.83–0.97	0.59–0.66	0.81–0.85	2.69–3.15	1.62–1.98	2.75–3.24
	0	≤ 1.24	≥ 1.07	≥ 2.41	≥ 4.21	≤ 0.82	≤ 0.58	≤ 0.80	≤ 2.68	≤ 1.61	≤ 2.74

CV: coefficient of variation, AC: autocorrelation coefficient, VT: vertical, ML: mediolateral, AP: anteroposterior, HR: harmonic ratio

Results of the factor analysis with promax rotation are presented in Table 3. This analysis yielded four distinct but correlated factors explaining 57.1% of the variance. The first factor accounted for 30.1% of the variance in the C-GAITS score and loaded on the AC for all directions. We labeled this factor the “regularity” factor. The second factor explained 11.3% of the variance and loaded on variables reflecting gait pace (i.e., walking speed and mean stride time) and was termed the “pace” factor. The third factor accounted for 9.9% of the variance and loaded on the CVs of stride time and swing time. We labeled this factor the “variability” factor. The fourth factor accounted for 5.9% of the variance and loaded on the HRs in all directions. We labeled this factor the “smoothness” factor. The correlation coefficients between the four subscales were above 0.20, except for the correlation between the pace factor and the variability factor (*r* = 0.19; Table 2). The highest correlation was observed between the regularity factor and the variability factor (*r* = 0.55).

**Table 3:** Factor loadings, proportion of variance, and factor correlation matrix for the 10 gait parameters included in the factor analysis

	Factor 1	Factor 2	Factor 3	Factor 4
	(Regularity)	(Pace)	(Variability)	(Smoothness)
Walking speed	0.01	**0.79**	0.01	0.00
Mean stride time	0.00	**0.80**	−0.05	0.01
CV of stride time	0.12	−0.06	**0.81**	−0.06
CV of swing time	−0.09	0.02	**0.65**	0.11
AC-VT	**0.77**	0.20	0.05	− 0.07
AC-ML	**0.83**	−0.07	−0.13	0.17
AC-AP	**0.83**	−0.08	0.11	−0.07
HR-VT	0.01	0.05	0.18	**0.51**
HR-ML	0.09	−0.08	−0.14	**0.69**
HR-AP	−0.07	0.08	0.14	**0.56**
% of variance	30.1	11.3	9.9	5.9
Factor correlation matrix				
Factor 2	0.29	1		
Factor 3	0.53	0.22	1	
Factor 4	0.23	0.21	0.33	1

The variables presented in boldface signify variables that loaded on each of the four factors above 0.5

CV: coefficient of variation, AC: autocorrelation coefficient, VT: vertical, ML: mediolateral, AP: anteroposterior, HR: harmonic ratio

**Fig. 1:**
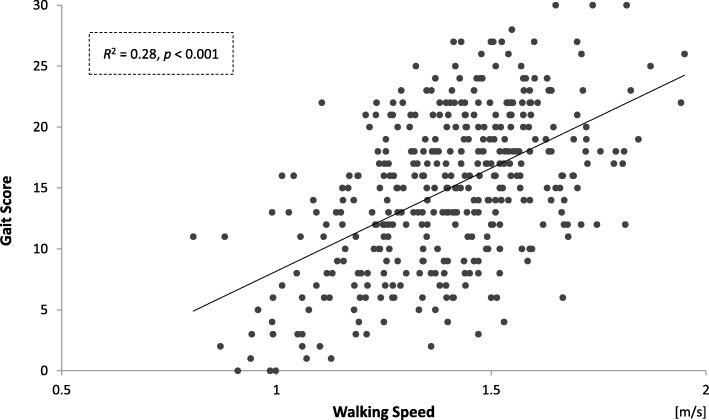
Single linear regression analysis between total C-GAITS score and walking speed

The internal consistency (Cronbach’s alpha coefficient) of the C-GAITS score was 0.77. The results of the single linear regression analysis between total C-GAITS score and walking speed are shown in Fig. 1. A higher total C-GAITS score was associated with faster walking speed (adjusted *R*^2^ = 0.28). The significant association remained in the linear regression analysis between walking speed and total C-GAITS score when walking speed score was excluded from the calculation of the C-GAITS score. In the results from the bivariate comparisons, total C-GAITS score was significantly associated with age group (mean ± SD = 13.2 ± 6.7 for older-old adults vs. 15.6 ± 6.2 for younger-old adults, *p* = 0.002), lower extremity strength (mean ± SD = 12.5 ± 6.5 for those with impaired lower-extremity strength vs. 15.3 ± 6.3 for those with normal lower-extremity strength, *p* = 0.007), balance function (mean ± SD = 11.7 ± 6.5 for those with impaired balance vs. 15.4 ± 6.2 for those with normal balance, *p* <  0.001), fall history in the previous year (mean ± SD = 13.5 ± 7.4 for fallers vs. 15.3 ± 6.1 for non-fallers, *p* = 0.04), and FoF (mean ± SD = 12.9 ± 6.6 for the FoF group vs. 15.7 ± 6.2 for the non-FoF group, p <  0.001; Table 4). Moreover, the variables associated with each of the subscales differed (Table 4). Significantly lower regularity scores were observed among those with impaired balance (*p* = 0.001), fallers (p = 0.007), and those with FoF (*p* = 0.048). Significantly lower pace scores were seen among older-old adults (p <  0.001), those with impaired lower-extremity strength (*p* = 0.01), those with impaired balance (p = 0.04), and those with FoF (p = 0.002). Significantly lower variability scores were observed among older-old adults (*p* = 0.03) and those with impaired balance (p = 0.04). Significantly lower smoothness scores were seen among older-old adults (p = 0.04), those with impaired lower-extremity strength (p = 0.01), and those with FoF (*p* = 0.003).

**Table 4:** Associations of total C-GAITS score and subscale scores with age group, lower extremity strength, balance function, fall history, and fear of falling

Variable	Gait score		Regularity index		Pace index		Variability index		Smoothness Index	
	mean ± SD	*p-*value	mean ± SD	*p-*value	mean ± SD	*p-*value	mean ± SD	*p-*value	means ± SD	*p* value
Older-old adult (aged ≥75 years)	13.2 ± 6.7	**0.002**	4.2 ± 3.1	0.33	2.3 ± 2.0	**< 0.001**	2.6 ± 1.9	**0.03**	4.0 ± 2.5	**0.04**
Younger-old adult	15.6 ± 6.2		4.6 ± 2.9		3.2 ± 2.0		3.2 ± 2.0		4.7 ± 2.6	
Impaired lower-extremity strength (5CS > 11.1 s)	12.5 ± 6.5	**0.007**	4.0 ± 3.2	0.22	2.3 ± 1.8	**0.01**	2.37 ± 2.1	0.35	3.6 ± 2.5	**0.01**
Normal lower-extremity strength	15.3 ± 6.3		4.5 ± 2.9		3.1 ± 2.0		3.0 ± 2.0		4.6 ± 2.5	
Impaired balance (tandem test < 3.0 s)	11.7 ± 6.5	**< 0.001**	3.1 ± 2.8	**0.001**	2.4 ± 2.2	**0.04**	2.4 ± 1.9	**0.04**	3.9 ± 2.6	0.11
Normal balance	15.4 ± 6.2		4.7 ± 2.9		3.1 ± 2.4		3.1 ± 2.0		4.6 ± 2.5	
Faller	13.5 ± 7.4	**0.04**	3.6 ± 3.0	**0.007**	2.6 ± 2.1	0.08	3.0 ± 1.0	0.95	4.3 ± 2.6	0.48
Non-faller	15.3 ± 6.1		4.7 ± 2.9		3.1 ± 2.0		3.1 ± 2.1		4.5 ± 2.6	
FoF	12.9 ± 6.6	**< 0.001**	4.0 ± 3.0	**0.048**	2.4 ± 2.1	**0.002**	2.7 ± 2.0	0.07	3.8 ± 2.4	**0.003**
No FoF	15.7 ± 6.2		4.7 ± 2.9		3.2 ± 2.0		3.1 ± 1.9		4.7 ± 2.6	

*P-*values were calculated using unpaired *t-*tests

C-GAITS score: Comprehensive Gait Assessment using InerTial Sensor, 5CS: five-chair-stand test, FoF: fear of falling, SD: standard deviation, tandem test: tandem stand test

## Discussion

We developed the C-GAITS score to incorporate the scores derived from multiple gait parameters, using acceleration and angular velocity data measured with inertial sensors attached to the heel and lower trunk. An exploratory factor analysis showed that the C-GAITS score had four factors: “regularity,” “pace,” “variability,” and “smoothness.” These factors explained 57.1% of the variance. The internal consistency of the score was found to be good. A moderate correlation was observed between the score and walking speed; 28% of the variance of the score was explained by walking speed. Additionally, lower C-GAITS score was significantly associated with older age, poorer performance (impaired lower-extremity strength and balance), fall history in the previous year, and FoF. These results indicate that the C-GAITS score has appropriate construct validity (structural validity and hypothesis testing) for comprehensively assessing gait performance among community-dwelling older adults.

The strength of the current study is that it showed that the C-GAITS score, obtained by accumulating scores derived from multiple gait parameters, had good validity for hypothesis testing. Older adults with lower physical function and higher fall risk exhibited lower C-GAITS scores, which indicated poor values for multiple gait parameters. These results are supported by previous research. Some previous studies reported that more than one gait parameter derived from trunk acceleration in all three directions was associated with lower physical function or falling risk [13, 14, 37]. For example, Moe-Nilssen et al. reported that frail older adults had lower values of AC-VT, AC-ML, and AC-AP, compared with fit older adults [13]., and Menz, et al. reported that older adults with high falling risk exhibited lower CVs of step time, HR-VT, HR-ML, and HR-AP, compared with those with low risk [14]. However, the subjects in these previous studies tended to be selected using convenience sampling, and there were large differences in functional level between the groups. In contrast, other studies showed that a parameter from one direction was the only associated factor [16–18, 23, 38]. In a previous 1-year prospective study, we found that only HR-VT predicted falling [18]. In another previous study, we found that only HR-ML was associated with FoF [16]. The samples in these studies tended to be community-based, and the differences in functional level between the groups were relatively small. Taken together, the results of the current study and the abovementioned findings from previous work may indicate that older adults with greater functional decline exhibit low values on a larger number of gait parameters and that older adults with less functional decline exhibit low values on fewer gait parameters. Overall, these results might provide a new orientation for methods using inertial sensors in gait assessment.

In the single linear regression analysis, the total C-GAITS score was moderately correlated with walking speed (*R*-squared = 0.28), which is a representative marker of gait performance. This result indicates that the C-GAITS score is valid for the assessment of gait performance. However, 72% of the variance of the score was not explained by walking speed. Clinically, it is important to assess not only walking speed but also “quality of gait movement.” In fact, several studies have reported that “quality of gait movement” parameters were associated with several kinds of functional declines, independent of walking speed, among older adults [16–18, 39]. The C-GAITS score includes parameters to assess regularity, variability, and smoothness, and some parts of the residual variance may be explained by these parameters. Hence, gait performance can be represented comprehensively by the C-GAITS score.

Exploratory factor analysis showed that the C-GAITS score had four factors, and each factor included the parameters from a similar calculation expression. The “regularity” factor included ACs in all three directions. AC represents the “stride-to-stride” regularity of lower trunk acceleration. The “smoothness” factor included HRs in the three directions, where HR represents the smoothness of lower trunk acceleration “within stride.” The “pace” factor included spatio-temporal gait parameters in the direction of travel. The “variability” factor included the CVs of stride and swing time, which represent the stride-to-stride variability of lower extremity movement. Thus, the factor analysis results can be considered reasonable. Several previous studies reported other models of gait domains based on the results of factor analysis of computerized walkway measurements [40, 41]. Several factors similar to ours were included in their results (e.g., pace factor or variability factor). The results of the present study add value to models of gait domain because we measured not only parameters from foot movements but also parameters from trunk movements during gait. In addition, we developed subscale scores based on the factor analysis, and the each of these was related to different clinical variables. These results suggest that the subscale scores provide deeper information on gait performance and that the subscales are useful for gait assessment. It is possible that efficient intervention programs to improve gait functions could be developed using the results of factors related with each of the subscales. To clarify these factors, further studies are needed.

The C-GAITS score was calculated based on the quartiles for each gait parameter by gender, and we presented the resulting values as reference data for community-dwelling older adults. In the past, several studies have reported reference values of gait parameters using inertial sensors [6, 7, 15]., and our values were comparable but slightly higher than those found in past studies. The current study used quartile values from a large sample of older adults for scoring, and the scores were found to be significantly related to functional declines. These results suggest that our reference data provide clinically useful values for gait assessment, and data obtained in the future can be evaluated accordingly. Additionally, we presented the reference values by gender, and differences were observed between men and women on several gait parameters (stride time, AC-ML, HR-ML, and HR-AP). These parameters have previously been reported to be associated with gender [9, 42, 43]. The reference data presented in this study can thus be considered reasonable. Therefore, the C-GAITS score will be highly useful in clinical settings—not only for clinicians but also for assessment subjects—as a tool to quickly assess whether there are gait abnormalities and which gait parameters are especially low.

Several limitations should be considered in this study. First, the validation of the C-GAITS score was ascertained only for the original sample used to develop the score. Additionally, our participants are community-dwelling older adults who were able to come independently to the place where measurements were taken, suggesting that they might be well-functioning older adults (sampling bias). Further studies are warranted to confirm the external validity of the score for community-dwelling older adults, including those at various stages of health and functionality. Another limitation was that a limited number of gait parameters were used to develop the C-GAITS score, although there are many gait parameters measured using inertial sensors (e.g., root mean square, approximate entropy, Lyapunov exponents, peak amplitude variability, and symmetry index) [44–47]. In our view, examining these additional gait parameters would be a fruitful next step for future research. In the present study, we chose 10 gait parameters as the components of the C-GAITS score; we selected parameters that were commonly reported in existing studies, were easier to understand, and were simpler to calculate. Therefore, we believe that the C-GAITS score will be easily accepted in clinical fields. In addition, the gait assessment using inertial sensors was conducted only once for each subject. Although a number of past studies assessed gait performance from a single walking trial [14–18, 38, 48, 49]., several studies have reported that reliability is increased by conducting additional walking trials and calculating gait parameters from more strides. Thus, in future studies, assessing gait twice or more per subject or using a higher number of strides would lead to better reliability [22, 50, 51].

## Conclusions

We developed a scoring method that uses inertial sensors to comprehensively assess gait performance among community-dwelling older adults: the C-GAITS score, which incorporates the scores from various gait parameters. Good internal consistency was observed, and the appropriate construct validity of this score was confirmed by the results of an exploratory factor analysis and hypothesis testing examinations. The C-GAITS score may be useful in clinical settings because of its convenience, ease of use and interpretation, and capability of capturing functional decline among older adults.

## Abbreviations

5CS: Five-chair-stand test
AC: Autocorrelation coefficient
AP: Anteroposterior
C-GAITS score: Comprehensive Gait Assessment using InerTial Sensor score
CV: Coefficient of variation
FoF: Fear of falling
HR: Harmonic ratio
ML: Mediolateral
tandem test: Tandem balance test
VT: Vertical
